# Targeted Toxins in Brain Tumor Therapy

**DOI:** 10.3390/toxins2112645

**Published:** 2010-11-01

**Authors:** Yan Michael Li, Walter A. Hall

**Affiliations:** Department of Neurosurgery, State University of New York Upstate Medical University, Syracuse, New York 13210, NY, USA; Email: liy@upstate.edu

**Keywords:** targeted toxin, immunotoxin, cytotoxin, diphtheria toxin, pseudomonas exotoxin

## Abstract

Targeted toxins, also known as immunotoxins or cytotoxins, are recombinant molecules that specifically bind to cell surface receptors that are overexpressed in cancer and the toxin component kills the cell. These recombinant proteins consist of a specific antibody or ligand coupled to a protein toxin. The targeted toxins bind to a surface antigen or receptor overexpressed in tumors, such as the epidermal growth factor receptor or interleukin-13 receptor. The toxin part of the molecule in all clinically used toxins is modified from bacterial or plant toxins, fused to an antibody or carrier ligand. Targeted toxins are very effective against cancer cells resistant to radiation and chemotherapy. They are far more potent than any known chemotherapy drug. Targeted toxins have shown an acceptable profile of toxicity and safety in early clinical studies and have demonstrated evidence of a tumor response. Currently, clinical trials with some targeted toxins are complete and the final results are pending. This review summarizes the characteristics of targeted toxins and the key findings of the important clinical studies with targeted toxins in malignant brain tumor patients. Obstacles to successful treatment of malignant brain tumors include poor penetration into tumor masses, the immune response to the toxin component and cancer heterogeneity. Strategies to overcome these limitations are being pursued in the current generation of targeted toxins.

## 1. Introduction

In 1906, Paul Ehrlich introduced the concept of targeting cancer cells with a “magic bullet”. His magic bullet consisted of using tissue-specific carriers to deliver toxic agents to neoplastic tissues [1,2]. The development of modern cancer therapy is based on discovering and designing drugs that target cancer specific pathways with minimal side effects. Targeted toxins, also called immunotoxins or cytototoxins, are recombinant molecules that specifically bind to surface antigens or receptors overexpressed in cancer, including tumor and endothelial cells [3]. These recombinant proteins consist of a specific antibody or ligand coupled to a toxin protein. The targeted toxins bind to a surface antigen or receptor overexpressed in cancer, such as the epidermal growth factor receptor, transferrin receptor, interleukin-13 or interleukin-4 receptor. Antibodies have been used for targeting chemotherapeutic drugs, toxins, enzymes and radionuclides, with the aim of developing targeted therapies for cancer. Antibodies in the therapy of cancer have evolved from mouse antibodies, to chimeric antibodies, to humanized antibodies. Meanwhile, due to poor penetration of toxins chemically conjugated to monoclonal antibodies (mAbs) [4,5], growth factors or cytokines linked to toxins were also used for targeted cancer therapy and they have been rapidly advanced into clinic trials. The toxin part of the molecule in all clinically used cytotoxins is modified from bacterial or plant toxins, in which the cell recognition domain is replaced with a new targeting moiety from an antibody or carrier ligand. Targeted toxins have the advantage of being highly cytotoxic and easy to manipulate by genetic engineering methods [2,6,7]. 

Malignant brain tumors such as the glioblastoma multiforme (GBM) are highly lethal tumors and the life expectancy for patients with GBM under the current standards of care is on average 14 months from diagnosis despite maximal therapy with chemotherapy and radiation therapy [8,9]. The poor clinical prognosis associated with malignant brain tumors has led investigators to seek new, innovative methods of treatment. Targeted toxins are extremely cytotoxic to malignant GBM cell lines *in vitro*. Animal studies have shown prolongation of survival and complete tumor regression when targeted toxins were administered by a variety of routes [7,10,11]. The promising results seen *in vivo* have formed the basis for proceeding with clinical trials in humans with malignant brain tumors and leptomeningeal neoplasia, in which these agents are administered directly into the tumor or intrathecally, respectively. To date, in these clinical trials, targeted toxins have been delivered safely without significant neurological toxicity, and cytological analysis of cerebrospinal fluid and radiological findings have shown evidence of a therapeutic response. These studies have confirmed the existence of a therapeutic window between normal brain tissue and malignant cells that can be exploited with targeted therapy directed against cancer specific receptors. The successful delivery of targeted toxins directly into malignant brain tumors has established this route of administration as both practical and feasible. 

This review summarizes the characteristics of target toxins and the key findings of the important clinical studies with targeted toxins in malignant brain tumor patients. Obstacles to the successful treatment of malignant brain tumors include poor penetration into tumor masses and the immune response to the toxin component. Strategies to overcome these limitations are being pursued. An outlook into future areas of development of targeted toxins will be discussed.

## 2. Toxins

The toxins used in most clinical immunotoxin or cytotoxin construction are made by bacteria or plants. They are very potent in small amounts delivered by these organisms, after natural selection over millions of years. Though structurally and evolutionarily different, Diphtheria toxin (DT) and *Pseudomonas aeruginosa* exotoxin A (PE) share similar properties of protein synthesis inhibition either by modifying elongation factor-2 or by directly inhibiting the ribosome [12]. Once attached to the overexpressed antigens or receptors on cancer cells, the toxin is endocytosed and transferred via an endosome to either a lysosome or the Golgi apparatus. The toxin and carrier ligand are then separated, allowing the toxin to inhibit protein synthesis. Immunotoxins can inactivate over 200 ribosomes or elongation factor-2s per minute. Furthermore, other mechanisms are also involved for toxins to disrupt the host cell function; for example, AB5 subtilase cytotoxin produced by pathogenic bacteria, such as Shiga toxigenic *Escherichia coli* (STEC), cleaves the essential endoplasmic reticulum chaperone protein BiP/GRP78, which is key for cell survival [13,14]. A single immunotoxin can kill a cancer cell as compared to 10^5^ molecules of a chemotherapeutic drug that are needed to kill one cancer cell. So these toxins are much more potent when compared to traditional chemotherapeutic drugs.

Most toxins are polypeptides with several domains: a cell recognition chain, which binds to the receptors on the surface of the target cell; a translocation chain, which enables the toxin to cross a membrane to reach the cytosol where essential cell machinery is located; and an inactivation domain, which inactivates some vital cellular process and causes cell death [2,3]. To make an immunotoxin, the cell recognition domain is replaced with a new recognition moiety. The most commonly used toxins in the clinical trials are two bacterial toxins: Diphtheria toxin and *Pseudomonas aeruginosa* exotoxin A [15]. 

Diphtheria toxin is a 62 kDa protein secreted by *Corynebacterium diphtheria* [16,17]. The single polypeptide chain must be enzymatically nicked at an arginine-rich site for the A and B chain to be activated against human cells. Diphtheria toxin (DT) has a cell-binding domain at the *C* terminus (amino acids 482–539) and the A chain with ADP-ribosylation activity at the *N* terminus. The A chain catalyzes the transfer of adenosine diphosphate (ADP)-ribose to EF-2, preventing the translocation of peptidyl-t-RNA on ribosomes, thereby blocking protein synthesis and subsequently killing the cell [18,19,20]. A natural ligand for DT on the cell membrane is the heparin-binding epidermal growth factor (EGF)-like precursor [21]. DT undergoes internalization, disulfide bond reduction and proteolytic activation after cell binding, but translocation into the cytoplasm occurs directly from the acidic endocytic compartment. Recombinant DT is made by replacing the *C* terminal cell-binding domain with a ligand that binds to a growth factor receptor or the Fv fragment of an antibody. The native DT protein consists of 535 amino acids. Variable truncation of the binding segments resulting in 389 and 486 amino acid length toxin conjugates has resulted in the formation of toxins DAB389 and DAB486, respectively [12]. These modified DTs are unable to enter a cell without selective uptake of their carrier ligand by a receptor. Another modification of DT involves substitution of two amino acids in the B chain resulting in a new molecule cross-reacting material-107 (CRM-107) [22,23,24]. This modification reduces the non-specific binding of DT to human cells by 8000-fold, thus increasing the toxin’s tumor-specificity 10,000-fold.

*Pseudomonas aeruginosa* exotoxin A is a single peptide with three functional domains: domain Ia is the *N* terminal and cell-binding domain; domain II has translocation activity; and domain III is the *C* terminal and catalyses the adenosine diphosphate (ADP)-ribosylation that inactivates EF-2, which further blocks protein synthesis and causes cell death. PE binds to the low density lipoprotein receptor-related protein (LRP1), also known as alpha-2-macroglobulin receptor or CD91, which is expressed in the plasma membrane of human cells and is then internalized through clathrin-mediated endocytosis. A 37 kDa fragment (amino acids 280–612) from the *C* terminus is released after proteolytic cleavage between amino acids 279 and 280 and reduction of the disulfide bond at amino acids 265 and 287. The fragment is transported to the endoplasmic reticulum, translocated to the cytoplasm, and then inactivates EF-2 [25,26]. The genetic excision of domain Ia results in a molecule termed PE 40 which retains its translocation function and EF-2 inhibition properties but is unable to kill human cells [25,27]. Furthermore, removal of the Ia domain should in turn decrease the hepatoxicity of PE immunotoxins that is due to residual binding of domain Ia to the hepatocyte. A genetically engineered PE molecule PE38KDEL, has amino acids 253–364 linked to amino acids 381–608 with a change in the carboxyl end of PE (KDEL) to increase cytotoxic activity [28,29]. PE38KDEL has been fused with a targeting moiety such as the antibody Fv portion, a growth factor, or cytokine and found to have a much higher affinity for binding to cancer cell lines than the native PE immunotoxin, and was much more toxic to malignant cells [30,31].

## 3. Clinical Trials in Brain Cancer

Immunotoxins were first shown to be potent cancer cell killers in the early 1970s. Initially, unmodified toxins were injected into the topical forms of refractory metastatic cancers in early clinical studies [10,32]. Afterwards, clinical trials investigating the efficacy and toxicity of immunotoxins in treating a wide variety of hematologic malignancies have made significant progress both with peripheral blood involvement such as in leukemias and malignancy outside the vasculature such as Hodgkin’s lymphoma and multiple myeloma [3,33]. Solid tumors including brain tumors are relatively resistant to immunotoxin treatment because of decreased access to the immunotoxin and a relatively intact immune system. The first report demonstrating the efficacy of immunotoxins against primary CNS tumor cell lines was not published until 1987 [17]. The first generation of immunotoxins conjugated the toxins directly to the Fc portion of a mAb. The clinical results were unremarkable. The second generation of immunotoxins conjugated the toxins to Fab’s or synthesized fusion proteins. The clinical results were much more promising, but still did not represent a definitive treatment modality [32]. Clinical and some promising preclinical studies in brain tumors are summarized below and some major features are discussed (Table 1).

**Table 1:** Targeted toxins used against brain tumors.

Immunotoxin	Toxin used	Target antigen	Administrative route	Clinical trial phase	Number and type of Tumor	Outcome	Adverse Effect	References
IL-4(38-37)-PE38KDEL	(38-37) PE38KDEL	IL-4R	Intratumoral (CED)	I/II	31 (25 GBM and 6 AA)	Median survival 8.2 months; Six month survival was 52%.	Headache, seizure, weakness, dysphasia, Hydrocephalus	[31,34,35]
IL13-PE38QQR	PE38QQR	IL-13R	Intratumoral (CED)	I/II/III	Phase II, 51 (46GBM, 3AA, other 2); Phase III, 296 recurrent GBM	Infusion MTIC was 0.5 µg/mL; up to 6 d well tolerated; Median survival 42.7 weeks (95% CI, 35.6–55.6) for GBM in phase II, and 36.4 weeks in phase III, comparable to Gliadel Wafer.	Headache, dysphasia, seizure, weakness, pulmonary embolism	[36,37,38]
TP-38	PE-38	TGF-α	Intratumoral (CED)	I	20 (17 GBM, other 3)	Median survival 28 weeks (95% CI, 4.1–45.1).	Hemiparesis, fatigue, headache, dysphasia	[15,39]
Tf-CRM107	DT-CRM107	Tf	Intratumoral (CED)	I/II	44 (GBM, AA)	Median survival 37 weeks, (95% CI, 26–49); 5/34 CR, 7/34 PR, response rate 35% (95% CI, 20–54; p < 0.0001).	Seizure, cerebral edema	[40]

GBM: Glioblastoma Multiforme; AA: Anaplastic Astrocytoma; TGF: transforming growth factor; CED: convection-enhanced delivery; MTIC: maximum-tolerated infusate concentration; CI: confidence interval; Tf: transferrin; CR: complete response; PR: partial responders; RR: radiographic response.

### 3.1. Pseudomonas exotoxin-based immunotoxins and cytotoxins

#### 3.1.1. IL4-PE

Interleukin-4 (IL-4) is a pleiotropic cytokine which is primarily produced by Th2-type T lymphocytes, mast cells, and basophils [41]. Normal cells such as B cells, endothelial cells, microglia, and astrocytes express low levels of IL-4 receptors [41,42]. Human malignant glioma cell lines and malignant astrocytic tumor specimens derived from surgical samples have been shown to overexpress high-affinity IL-4 receptors (IL-4R) *in vitro* and *in situ* [43]. The significance of IL-4R expression on malignant glioma cells is still unclear. However, IL-4 has been reported to mediate functional effects in several solid tumor cell lines, including inhibition of cell proliferation, and induction of signal transduction through the JAK/STAT pathway [44]. Recombinant fusion protein IL-4(38-37)-PE38KDEL, cpIL4-PE or NBI-3001, which for simplicity is called IL4-PE in this review, was constructed and expressed, consisting of a binding ligand, circularly permuted IL-4 and a mutated form of Pseudomonas exotoxin. Recombinant IL4-PE is highly and specifically cytotoxic to glioma cell lines *in vitro*, while it is less cytotoxic to hematopoietic and normal brain cells. In a nude mouse model, IL4-PE showed significant antitumor activity and partial or complete regression of small or large established human GBM tumors [45,46]. Preliminary clinical results suggested that IL4-PE can cause pronounced necrosis of recurrent GBM without systemic toxicity [46,47]. 

Based on these pilot studies of IL4-PE, an extended Phase I/II clinical trial was conducted to determine safety, tolerability, and efficacy of IL4-PE when injected directly into recurrent GBM by convection enhanced delivery (CED). Six of nine patients showed glioma necrosis as evidenced by decreased enhancement on MRI [31]. An open-label, dose-escalation trial of IL4-PE reported that a total of 31 patients with histologically verified supratentorial grade 3 and 4 astrocytoma and Karnofsky Performance Scores (KPS) ≥ 60 were assigned to one of four dose groups in a dose-escalation fashion (6, 9 and 15 μg/mL, a total volume of 40 mL or 100 mL). IL4-PE was administered intratumorally via stereotactically placed catheters. The overall median survival was 8.2 months with a median survival of 5.8 months for the GBM patients. Six-month survival was 52% and 48%, respectively. MRI showed areas of decreased signal intensity within the tumor consistent with possible tumor necrosis and decreased contrast enhancement immediately following treatment in many patients. Although tumor necrosis was not confirmed by biological examination, IL4-PE induced change in gadolinium enhancement representing positive tumor necrosis was confirmed by histological examination of tissues in several patients. No IL-4-PE could be detected in the plasma, No drug-related hematological or serum chemical changes was apparent in any patients; treatment-related adverse effects were limited to the CNS, with drug-related Grade 3 or 4 toxicity in 39% of patients [48]. IL4-PE delivered by CED was safe without systemic toxicity, however, the CNS toxicity observed was attributed to the volume of the infusion and/or nonspecific toxicity. One case reported long-term survival of three years in a patient with recurrent malignant glioma following intratumoral infusion of IL4-PE with a durable tumor response [35]. 

#### 3.1.2. IL13-PE

Interleukin-13 (IL-13), structurally similar to IL-4 and secreted by activated type 2 T cells and mast cells, is a pleiotropic lymphokine regulating inflammatory and immune responses [49]. This cytokine modulates human monocyte and B-cell functions but not T-cell function [50]. IL-13 binds to three chains (IL-13Ra1, IL-13Ra2, and IL-4Ra) and induces phosphorylation of STAT-6 by the Jak family [51,52]. IL-13 receptors are found to be overexpressed in solid tumor cells including GBM [53,54,55,56], renal cell carcinoma [56], and cancers of the prostate [57], ovary [58], and head and neck [59]. IL-13 has proven to be a useful ligand for therapy because, although it is overexpressed on many solid tumor cells including GBM cells, the only normal cells targeted are B cells and monocytes. IL-13 receptors (IL-13R) are tumor-specific, high-affinity targets that justify incorporating IL-13 into a targeted toxin as a promising strategy [53,54,55]. The recombinant fusion cytotoxin IL13-PE38QQR or cintredekin besudotox (CB), which is called IL13-PE for simplicity, was composed of IL-13 and a mutated form of PE [60,61]. IL13-PE is specifically cytotoxic to glioma cell lines *in vitro* and has significant antitumor activity and partial regression of the established human GBM tumors, while it is less cytotoxic to normal human brain cells. 

IL13-PE has been tested in four Phase I/II clinical trials and was administered intracranially using CED [37,38,62] for patients with recurrent or progressive resectable supratentorial WHO grade 3/4 malignant glioma. The drug was delivered through catheters placed either directly into the tumor mass or in the peritumoral region after resection of the lesion for 96 hours at an infusion rate of 0.75 mL/h divided between 1 to 3 catheters. The CED of IL13-PE was fairly well tolerated. The maximum tolerated intraparenchymal concentration was 0.5 μg/mL for up to six days and tumor necrosis was observed at this concentration. Catheter placement was important for optimal drug distribution. Overall median survival for GBM patients was 42.7 weeks (95% confidence interval [CI], 35.6–55.6) and 55.6 weeks (95% CI, 36.1–74.3) for patients with optimally positioned catheters with patient follow-up extending beyond five years [38]. 

The Phase III Randomized Evaluation of CED of IL13-PE compared to Gliadel Wafer (GW) with Survival Endpoint Trial, known as the PRECISE Trial, in patients with initial recurrence of GBM has recently been completed. Patients were randomized 2:1 to receive IL13-PE or GW. IL13-PE (0.5 mg/mL, 0.75 mL/h over 96 hours) was administered via 2–4 intraparenchymal catheters placed in areas at greatest risk for infiltrating disease or in the vicinity of any residual, solid, contrast-enhancing disease 2–7 days after tumor resection. GW (3.85%/7.7 mg carmustine per wafer; maximum 8 wafers) were placed immediately after tumor resection. There were 296 patients enrolled at 52 centers. The primary endpoint was overall survival from the time of randomization. Secondary and tertiary endpoints were safety and health-related quality-of-life assessments. Median survival was 36.4 weeks for IL13-PE and 35.3 weeks for GW (P = 0.476, hazard ratio 0.89; 95% CI 0.67–1.18). For the efficacy evaluable population, the median survival was 45.3 weeks for IL13-PE and 39.8 weeks for GW (P = 0.310, hazard ratio 0.81; 95% CI 0.67–1.18). The adverse-events profile was similar in both groups, except that pulmonary embolism was 8% for IL13-PE *vs.* 1% in the GW group (P = 0.014). Although there is no statistical difference in the survival between the two treatment groups, this trial was the first randomized phase III evaluation of a targeted toxin administered via CED with an active comparator in patients with malignant glioma [36].

#### 3.1.3. TP-38

The epidermal growth factor receptor (EGFR) is one of four known members of the human epidermal growth factor receptor (HER) tyrosine kinase family [63]. The receptor is a 170 kDa transmembrane protein with extracellular receptor domain, which upon binding to its ligand, EGF or transforming growth factor-α (TGF-α), results in receptor dimerization [64]. EGFR has been found to be amplified or overexpressed in a large proportion of GBM cells [65,66]. Gene amplification with loss of feedback inhibition and mutation play significant roles. Furthermore, it has been shown in some cancers that overexpression of EGFR correlates to poor outcome. A number of studies have addressed the prognostic value of EGFR expression and mutation in gliomas [65,66]. Immunotoxin TP-38 is a 43.5 kDa recombinant protein fusing PE-38 with TGF-α which is specifically targeted the EGFR [30]. The phase I clinical trial of TP-38 targeted EGFR in patients with a KPS score ≥ 60 with recurrent primary or metastatic malignant brain tumor using CED. Twenty patients were enrolled and stratified for dose escalation (25, 50 and 100 ng/mL, a total volume of 40 mL). Radiographic responses were defined as before [67] on consecutive contrast-enhanced MR or CT at least four weeks apart, with clinical neurological stability or improvement and no increase in steroid dose. Two dose limiting neurologic toxicities were seen, including a grade 3 hemiparesis and grade 4 constitutional symptoms (fatigue). Median survival after TP-38 was 28 weeks (95% CI, 4.1–45.1). For patients with residual disease, median survival was 20.1 weeks (95% CI, 16.7–110.0), while for those without radiographic evidence of residual disease, median survival was 33.0 weeks (95% CI, 0–170.4). Two of 15 patients treated with residual disease demonstrated radiographic responses, including one patient with GBM who had a nearly complete response and remains alive >260 weeks after therapy. TP-38 delivered by CED was well tolerated with some durable radiographic responses [39]. However, the potential efficacy of drugs delivered by this technique may be severely influenced by ineffective infusion in many patients as evidenced by imaging the co-infused ^123^I-albumin [15].

### 3.2. Diphtheria toxin-based immunotoxins and cytotoxins

The US Food and Drug Administration (FDA) approved the first targeted toxin drug Ontak for cutaneous T-cell lymphoma in 1999 after successful Phase I, II and III trials [68]. ONTAK (DAB389IL-2) is a ligand fusion toxin consisting of the full-length sequence of the IL-2 gene fused to the enzymatically active and translocating domains of DT [69]. Other targeted DT toxins in development stages of either preclinical or clinical trials are discussed below.

#### 3.2.1. Tf-CRM107

The transferrin (Tf) receptor (TfR) is a transmembrane glycoprotein which mediates the cellular uptake of iron [70,71,72]. TfR expression is up-regulated in dividing cells compared to resting cells [72]. TfR have been shown to be expressed in high numbers on malignant tumors, making it an attractive candidate for selective immunotoxin targeting [73,74,75,76]. Transferrin-CRM107 (Tf-CRM107) is a conjugate protein of DT with a point mutation (CRM107) linked by a thioester bond to human Tf [77,78,79]. This conjugate exhibits potent cytotoxicity *in vitro* against mammalian cells expressing the TfR with activity at picomolar concentrations. 

Phase I clinical trial of Tf-CRM107 is a single center, dose-escalating single arm trial, in patients with malignant primary or metastatic brain tumors refractory to conventional therapy. The results demonstrated that Tf-CRM107, delivered via a high-flow convection method utilizing stereotactically placed catheters, produced tumor responses without severe neurologic or systemic toxicity [40]. Following Tf-CRM107 infusion, a ≥50% decrease in tumor volume showed on MRI occurred in nine of the 15 patients who could be evaluated (60%), including one patient having no evidence of tumor on MRI for 23 months after a single infusion of Tf-CRM107 into a progressing recurrent GBM and no tumor cells on the biopsies of the region of treatment at two and 10 months after treatment. 

Phase II study of Tf-CRM107 was a multicenter trial of intratumoral CED infusion of Tf-CRM107 for the patients with recurrent GBM or anaplastic astrocytoma (AA). Patients then received two Tf-CRM107 infusions (0.67 μg/mL, up to 0.40 mL/h, total volume of 40 mL) during a 4-10 week period. A complete response was defined as disappearance of all solid areas of enhancement. A partial response was defined as a ≥50% decrease in the enhancing volume of the treated tumor. Patients with <50% reduction in enhancing tumor volume or an increase in the tumor volume were considered non-responders. The results of a Phase II study also showed that Tf-CRM107 treatment resulted in a total of five complete responders (CR) and seven partial responders out of the 34 evaluable patients. The estimated proportion of complete or partial responders was 35% of evaluable patients (95% CI, 20–54%, p < 0.0001). The median survival time was 37 weeks (95% CI, 26–49 weeks). Thirteen of the 44 patients (30%) survived beyond 12 months from the time of the first treatment, and one patient survived 3.1 years. Symptomatic progressive cerebral edema occurred in eight of the 44 patients (14%) that was responsive to medical management. These data warranted a Phase III study as well as continued research in the field of targeted toxin therapy. Future directions of research has included optimizing Tf-CRM107 delivery to targeted brain regions and improving the treatment efficacy by combining this agent with other toxin conjugates targeted to different receptors. A phase III study commenced around 2005 but was discontinued because of a failure to demonstrate a positive therapeutic response. 

#### 3.2.2. DTAT and DTAT13

The roles of the serine protease urokinase-type plasminogen activator (uPA) and its receptor (uPAR) in glioma-cell invasion and neovascularization have attracted a lot of attention. uPA is produced as an inactive single-chain protein known as pro-uPA, which binds to uPAR and is activated by plasmin [80]. The expression of uPAR by human GBM cell lines contributes to their invasive capability [81,82]. uPAR is expressed at greater levels by anaplastic astrocytoma and GBM cells than by normal brain tissue or low-grade gliomas [83,84,85]. uPAR tends to be found at the leading margin of the tumor [81,82]. The recombinant fusion protein DTAT that targets uPAR and delivers the potent catalytic portion of DT has the advantage of simultaneously targeting both overexpressed uPAR on GBM cells and on tumor neovasculature [86,87]. The recombinant protein was highly selective for human GBM *in vitro* and *in vivo* and caused the regression of subcutaneous uPAR-expressing tumors with minimal toxicity to critical organs [87,88]. A bispecific immunotoxin DTAT13 was also synthesized in order to target simultaneously uPAR and IL-13 receptor expressing GBM cells [89]. DTAT13 is highly selective and synergistic for human GBM compared with DTAT and DTIL13 controls. DTAT13 caused the regression of small tumors and was able to target both GBM and the tumor vasculature with less toxicity than DTAT or DTIL13 [90,91].

### 3.3. Other Toxins

Ricin-based immunotoxins are probably some of the most frequently studied immunotoxins to date. Clinical trials using Ricin A chain conjugates as well as galactose binding site-blocked intact ricin conjugates started as early as 1994, primarily focusing on hematological malignancies [3,33,92]. For metastatic brain tumors, an early clinical trial using a human TfR MAb conjugated to ricin A chain (454A12-rRA) was administered intrathecally to patients with carcinomatous meningitis with doses ranging from 1.2 to 1200 µg [78,93]. A CSF inflammatory response manifesting with headache, vomiting, and mental status change, occurred at doses ≥120 µg. Four of the eight patients demonstrated a greater than 95% transient reduction in tumor cell counts in their CSF. One patient improved clinically, but none of the patients survived long term.

To avoid the immunogenicity associated with bacterial or plant toxins, human cytotoxic proteins such as ribonuclease or granzyme B have been used to target endothelial cells in tumors or tumor cells [94]. Furthermore, the expression of cancer-related proteases provides the opportunity to convert toxins into precursor toxins by replacing the furin cleavage site with a protease expressed in cancer cells. For example, the toxin is not active until cleaved by furin, so the furin site can be replaced by a site cleaved by urokinase using genetic mutation [95]. Several single-chain ribosome-inactivating proteins have also been used to make targeted toxins [96,97].

## 4. Current Status and Future Direction of Targeted Toxins

Several obstacles have influenced the therapeutic progress of immunotoxins in cancer treatment. The first concern is that immunotoxins target cell surface antigens that are highly expressed on the tumor cells but are also expressed on normal tissues, usually at a much lower level than on the tumor [2,7]. So bystander cell death can occur with an increase in either the dose or the rate of immunotoxin administration. Another example of nonspecific toxicity is vascular leak syndrome where there is fluid leakage from capillaries, a fall in the serum albumin level, fluid retention, edema and weight gain. This toxicity, owing to endothelial cell damage caused by the high concentration of immunotoxins, can usually be managed with adequate hydration, although severe vascular collapse has been observed at high doses of ricin-based immunotoxins [98,99]. The second concern is the limited access of tumor cells to targeted toxins. The diffusion rate of immunotoxins into brain tumors is affected by the blood-brain barrier [100], the high intra-tumor interstitial pressure in solid tumor [5,101], and local antigen binding [102]. The third disadvantage is that toxins are foreign proteins, and patients with solid tumors and normal immune systems may develop neutralizing antibodies which prevent retreatment [3,103]. There are multiple approaches to reduce the immunogenicity of immunotoxins, including genetic modification of key epitope recognized by T and/or B lymphocytes to generate anti-toxin antibodies without losing toxin activity. For example, seven major epitopes in the PE are recognized by B cells [104]. Mutation of these specific hydrophilic amino acids creates the new mutant PE38 proteins, which have significantly less immunogenicity but still retain full cytotoxic and anti-tumor activities [105,106]. The last and possibly the most important obstacle is the antigen or receptor heterogeneity that is due to the genetic heterogeneity of cancer, although cytotoxic effects of immunotoxins on brain tumor cell lines have been established, which means the antigens or receptors of the primary and metastatic tumors are not homogenous and are variable in density or structure [107]. For example, there is a discrepancy in receptor expression for TfR on the human medulloblastoma DAOY cell line *in vitro* and *in vivo* [108]. Furthermore, the EGFRvIII mutation is found in a large number of GBM and is the result of deletion of exons 2–7, with truncation of the extracellular portion of the protein and the subsequent inability to bind the ligand EGF, so that the immunotoxin with EGF as carrier ligand will not kill this population of GBM with EGFRvIII mutation [109]. 

Clinical responses to immunotoxins have mainly been observed in hematological malignancies and are not as common in solid tumors, including GBM [2,3,33]. This discrepancy may be explained by the fact that the tumor cells in the blood and bone marrow are easily accessible to the immunotoxin, while solid tumor cells such as GBM are not. A second reason for a difference in treatment response is that the immune system in hematological malignancies is impaired and damaged by previous chemotherapy administration so that anti-immunotoxin antibodies are not readily produced and therefore more than one cycle of treatment may be given. Furthermore, systemic toxicity can be caused by targeting the toxin to normal tissues that contain the same target antigen as the cancer cell. This is not a concern if immunotoxins are targeted to antigens on B- or T-cell malignancies, since normal B and T cells can be regenerated from antigen-negative stem cells. But it is a serious concern if solid tumors are targeted that have antigens present on vital organs such as the kidneys, liver or nerve cells, where the immunotoxin will kill these normal cells [110,111]. It is important to choose the target antigen or receptor, which is specifically overexpressed on the cancer cell, but not on normal cells.

The current generation of targeted toxins dates to the last several years where investigators have been engineering these drugs to bind to receptors but also to overcome two major hurdles: toxicity and immunogenicity. Further progress will depend on the identification of new antigenic targets on tumors and the production of less immunogenic immunotoxins so that patients can receive several treatment cycles. These agents may be particularly appropriate for patients with recurrent, resistant and widespread intracranial malignancy resistant to conventional therapy, including surgery, chemotherapy and radiotherapy.
